# A robust genomic signature for the detection of colorectal cancer patients with microsatellite instability phenotype and high mutation frequency#

**DOI:** 10.1002/path.4092

**Published:** 2012-10-12

**Authors:** Sun Tian, Paul Roepman, Vlad Popovici, Magali Michaut, Ian Majewski, Ramon Salazar, Cristina Santos, Robert Rosenberg, Ulrich Nitsche, Wilma E Mesker, Sjoerd Bruin, Sabine Tejpar, Mauro Delorenzi, Rene Bernards, Iris Simon

**Affiliations:** 1 Agendia NV, Amsterdam, The Netherlands; and Agendia Inc.Irvine, CA, USA; 2 Swiss Institute for BioinformaticsLausanne, Switzerland; 3 Netherlands Cancer InstituteAmsterdam, The Netherlands; 4 IDIBELL, Institut Catala d'Oncologia, L'Hospitalet de LlobregatBarcelona, Spain; 5 Klinikum Rechts der Isar, Technische Universität MünchenGermany; 6 Leiden University Medical CentreLeiden, The Netherlands; 7 University Hospital Gasthuisberg, Katholieke Universiteit LeuvenBelgium; 8 Département de Formation et Recherche, Centre Hospitalier Universitaire VaudoisFrance; 9 University of LausanneSwitzerland

**Keywords:** Colorectal cancer, microsatellite instability, deficient mismatch repair system, gene expression, mutation frequency, genomic signature, prognosis

## Abstract

Microsatellite instability (MSI) occurs in 10–20% of colorectal tumours and is associated with good prognosis. Here we describe the development and validation of a genomic signature that identifies colorectal cancer patients with MSI caused by DNA mismatch repair deficiency with high accuracy. Microsatellite status for 276 stage II and III colorectal tumours has been determined. Full-genome expression data was used to identify genes that correlate with MSI status. A subset of these samples (*n* = 73) had sequencing data for 615 genes available. An MSI gene signature of 64 genes was developed and validated in two independent validation sets: the first consisting of frozen samples from 132 stage II patients; and the second consisting of FFPE samples from the PETACC-3 trial (*n* = 625). The 64-gene MSI signature identified MSI patients in the first validation set with a sensitivity of 90.3% and an overall accuracy of 84.8%, with an AUC of 0.942 (95% CI, 0.888–0.975). In the second validation, the signature also showed excellent performance, with a sensitivity 94.3% and an overall accuracy of 90.6%, with an AUC of 0.965 (95% CI, 0.943–0.988). Besides correct identification of MSI patients, the gene signature identified a group of MSI-like patients that were MSS by standard assessment but MSI by signature assessment. The MSI-signature could be linked to a deficient MMR phenotype, as both MSI and MSI-like patients showed a high mutation frequency (8.2% and 6.4% of 615 genes assayed, respectively) as compared to patients classified as MSS (1.6% mutation frequency). The MSI signature showed prognostic power in stage II patients (*n* = 215) with a hazard ratio of 0.252 (*p* = 0.0145). Patients with an MSI-like phenotype had also an improved survival when compared to MSS patients. The MSI signature was translated to a diagnostic microarray and technically and clinically validated in FFPE and frozen samples. Copyright © 2012 Pathological Society of Great Britain and Ireland.

## Introduction

There are at least two recognized pathways of colorectal carcinogenesis (1). The most common pathway is a progressive model that involves stepwise accumulation of genetic alterations in several key oncogenes and tumour suppressor genes, such as KRAS, BRAF, TP53 and, importantly, the adenomatous polyposis coli (APC) gene (2, 3). These tumours account for approximately 85% of all sporadic disease and commonly display a chromosomal instability (CIN) phenotype that is associated with widespread structural alterations. A second class of colon tumours manifests a microsatellite instability (MSI) phenotype; these tumours typically display various insertions or deletions, most commonly in short tandem repeats, the so-called microsatellites (4). MSI is the molecular fingerprint of a deficient mismatch repair system. Approximately 15% of colorectal cancers (CRCs) display MSI, owing either to epigenetic silencing of MLH1 or to somatic or germline mutations in one of the mismatch repair genes MLH1, MLH3, MSH2, MSH6 or PMS2 (5). Consequently, the MSI phenotype is also referred to as the deficient MMR (dMMR) phenotype. MSI rates vary with tumour stage and, in the adjuvant setting, MSI patients have been associated with longer survival than patients with microsatellite-stable (MSS) tumours (6, 7). The deficiencies in MMR genes lead to loss of function of tumour suppressor genes and are associated with activating mutations in oncogenes such as BRAF (8).

Patients with MSI cancers might have different responses to chemotherapy compared to MSS patients (1, 9). The MMR involves the recognition and repair of incorrectly paired nucleotides during DNA replication. 5-Fluorouracil (5-FU)-based chemotherapy is the standard treatment for stage II and III CRCs after surgery, and the survival advantage associated with this treatment is about 10% (10). Data from patients with MSI and from cell lines with dMMR indicate that MSI promotes resistance to 5-FU treatment (1). However, results from clinical studies are conflicting. It seems that MSI patients with stage II cancer have no benefit from 5-FU treatment (11, 12), while stage III MSI patients might benefit from treatment, but this is predominantly seen in patients that have a germline predisposition (13). Evidence supporting the preferential efficacy of irinotecan in MSI tumours continues to emerge, but are still considered preliminary (14). Other studies have shown that MSI colorectal cancer might be specifically sensitive to compounds inhibiting the phosphatidylinositol 3-kinase (PI3K)–AKT–mammalian target of rapamycin (mTOR) pathway (15).

Considering the different prognosis and treatment response of MSI patients when compared to MSS patients, an accurate diagnosis is needed to facilitate appropriate treatment decisions. Today, several methods for the detection of MSI status are used. MSI can be detected by PCR amplification of specific microsatellite repeats. The presence of instability is determined by comparing the length of nucleotide repeats in tumour cells and normal cells. A consensus conference established a panel of microsatellite markers with appropriate sensitivity and specificity to diagnose MSI (16). This reference panel, known as the Bethesda panel, included five microsatellite loci: two mononucleotides (Bat25 and Bat26) and three dinucleotides (D5s346, D2s123 and D17s250) (17). Immunohistochemical analysis of MMR proteins is an alternative method to detect MSI in the clinical setting and complements the genetic testing of Lynch syndrome (18). Lack of expression of one or more of the MMR proteins is indicative of deficient MMR, and can help to determine which gene harbours a germline mutation or has been inactivated by another mechanism. However, traditional methods for determining MSI status might not identify all patients with a deficient mismatch repair system and other methods might be required for a more comprehensive detection (19).

As demonstrated by others (15, 20) and in this paper, patients with MSI have a very distinct gene expression pattern that allows the development of strong gene expression signatures. Pairwise comparisons between studies showed that 94–98% of genes have consistent changes in expression, even though samples were analysed on different platforms and in different studies (20). Here we describe the development and validation of a robust gene expression signature that identifies patients with MSI status, determined by standard methods (PCR, IHC) with high accuracy, and additionally identifies a group of MSS patients with a MSI-like phenotype. The signature was translated into a diagnostic test that can be used in fresh or FFPE material and can be performed in combination with other gene expression signatures (21, 22) for further classification of early-stage colon cancer patients.

## Methods

### Patients and samples

In this study, microsatellite instability was assessed in three patient cohorts that have been described previously: a development cohort (A) [22], a first independent validation cohort (B) (23) and a second independent cohort in the subset of the PETACC-3 gene expression dataset with complete MSI status information (cohort D) 24–26. The prognostic value of the developed MSI signature was assessed on cohort B combined with an additional set of samples with patient follow-up data but without hospital-based MSI assessment (cohort C). Patient and sample characteristics are shown in Table 1. All tissue samples were collected from patients with appropriate informed consent. The study was carried out in accordance with the ethical standards of the Helsinki Declaration and was approved by the medical ethical boards of the participating medical centres and hospitals.

**Table:** Patient characteristics

Cohorts	A	B	C	D	Total
	Development	Validation	Validation (prognosis)	Validation	
Patients (*n*)	276	132	131	625	1164
Tissue type	Fresh	Fresh	Fresh	FFPE	
Age					
< 70	157	84	60	529	830
≥ 70	119	48	71	96	334
Stage					
I	40	–	–	–	40
II	157	132	131	104	524
III	78	–	–	521	599
IV	1	–	–	–	1
Gender					
Male	165	74	66	382	687
Female	111	58	65	243	477
Location					
Left colon	143	76	56	391	666
Right colon	96	56	57	234	443
Rectum	37	–	10	–	47
Not available	–	–	8	–	8
Grade					
1	83	1	21	–	105
2	172	90	87	567*	916*
3	20	41	21	55*	137*
Not available	1	–	2	3	6
BRAF					
Activating mutation	24	18	13	46	101
Wild-type/unknown mutation	248	86	92	577	1003
Not available	4	28	26	2	60
Microsatellite stability					
MSI	29	31	–	70	130
MSS	247	101	–	555	903
Not available	–	–	131	–	131

* The PETACC3 dataset dichotomized the grade information by grouping stages 1 and 2, and 3 and 4, respectively.

### Hospital-based assessment for microsatellite instability (MSI)

For the development cohort (cohort A), fresh-frozen tumour samples from patients with colorectal cancer were collected (*n* = 276; Table 1). For 90 patients, 5 µm slides were immunohistochemically stained for the markers MLH1 and PMS2. For the remaining 186 patients and for all patients in validation cohort B (*n* = 132; Table 1) the MSI/MSS status was assessed by PCR amplification, following the standard protocol of the hospital and described in (21,22,26) and in Supplementary methods (see Supplementary material). Patients who had at least two microsatellite unstable markers were defined as MSI. A tumour with only normal markers was defined as microsatellite-stable (MSS). MSI assessment of the PETACC-3 samples (cohort D) was performed as described previously, using a standard panel of 10 mononucleotide and dinucleotide microsatellite loci by PCR amplification of normal/tumour DNA pairs (26). Irregularity in one marker (two in the PETACC-3 study) was defined as low-grade microsatellite instability (MSI-L); irregularity in more markers was defined as high-grade microsatellite instability (MSI) (27). Patients with MSI-L were classified as MSS for all analysis.

### Development and validation of a 64-gene signature associated with MSI status

RNA extraction, T7-based linear amplification, Cy-dye labelling and hybridization to Agilent arrays was performed as described previously (22). All tumour samples contained > 30% tumour cells. Samples were analysed against a common reference that was generated using a pool of 44 CRC samples. Gene expression measurements were normalized (Lowess normalization) and log-ratios were used for identification of genes that were associated with the MSI status of the tumours (based on two-sided Student's t-test). We used a 10-fold cross-validation (CV10) procedure that has been described previously (22, 28). The CV10 procedure was applied on the development cohort (*n* = 276) and repeated 1000 times to determine classification performance and for robust gene selection. During each CV10 round, genes were ranked by p value. The 64 genes (see Supplementary material, Table S1) with the highest frequency of appearance within the top-ranking genes in each of the 1000 CV loops were selected as the final set with the strongest MSI association (http://research.agendia.com/).

The 64 gene set was used to construct a nearest centroid-based classification method (cosine correlation); a MSI gene signature index for the individual samples was defined as the difference of the two correlations. Samples were classified within the MSI group if their index exceeded a predefined optimized threshold. This threshold was determined to reach a maximal overall accuracy (sum of sensitivity and specificity).

The 64-gene signature was validated on 132 independent CRC samples analysed in the same way as the development cohort, using the same microarray platform and threshold (cohort B, Table 1). Samples were classified as MSI if their index (the difference of the two correlations) exceeded the predefined optimized threshold. A second validation was performed on data from the PETACC-3 study comprising 625 colon tumour FFPE samples with known MSI status, of which 70 (11.2%) were MSI (cohort D, Table 1). As described previously (25), these 625 samples had been hybridized to a custom Affymetrix platform optimized for analysis of degraded RNA in FFPE samples. We could identify 58 of the 64 MSI signature genes. Read-out of the MSI gene signature index on the Affymetrix data was done in a similar fashion as for the first validation cohort. A receiver operating characteristic (ROC) curve was plotted and the area under the ROC curve (AUC) was calculated. Sensitivity and specificity were calculated based on the optimal overall accuracy, with a sensitivity of at least 90%.

**Figure 1:**
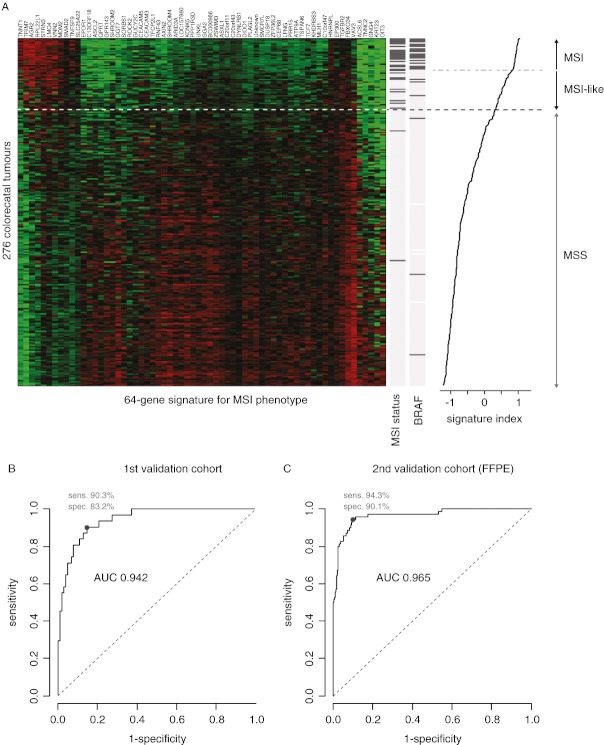
(A) A 64-gene expression signature for identification of colorectal cancer samples with MSI, MSI-like and MSS phenotypes. The MSI signature read-outs (index) are shown for 276 tumour samples (cohort A): red, relative up-regulation; green, down-regulation. Standard hospital-based MSI assessment is indicated in the middle bars, together with the *BRAF* V600E mutation status: light grey, MSS or *BRAF* wild-type, dark grey, MSI or *BRAF* mutation. (B) ROC curve and AUC of the signature read-out on validation cohort B. (C) ROC curve and AUC of the MSI signature on validation cohort D (PETACC-3 study). The optimal sensitivity and specificity (with a sensitivity of at least 0.9 and sum of sensitivity and specificity is maximal) is indicated in grey.

Besides the main binary classification of MSS and MSI samples, a secondary threshold was determined to subclassify MSI-like samples that were positive by MSI gene expression signature but typically classified as MSS by hospital assessment. Both thresholds for MSI and MSI-like classification were determined using the development cohort A only and are indicated in Figure 1A.

### Functional analysis of 64-gene signature

Functional analysis of the genes in the signature was performed by using the Database for Annotation, Visualization and Integrated Discovery (DAVID) software, v 6.7 (29). The enriched functional annotation clusters were calculated by DAVID through grouping enriched functional terms. The parameter set used had a similarity threshold of 0.4, multiple linkage threshold of 0.3 and an EASE parameter of 0.5. Only clusters larger than three functional terms were used.

### Investigation of mutation frequency

DNA fragment libraries were prepared using the TruSeq DNA Sample Preparation Kit (Illumina) and were hybridized to the SureSelect Human Kinome bait library according to the manufacturer's protocol (Agilent). Captured DNA samples were sequenced on a HiSeq 2000 (Illumina), using a 55 bp paired-end protocol. Sequence reads were aligned to the human genome (GRCh37/hg19) and unique pairs were used for variant calling. Candidate variants were identified using SAMtools and the following inclusion criteria were applied: minimum coverage 10; minimum variant count 5; a variant must be detected on both strands. Variants were assessed using the Ensembl variant effect predictor (v 62) to define those that were likely to impact protein coding sequences and to filter out germline polymorphisms. Matched germline DNA was sequenced for 19 of the 73 tumour samples and an additional 56 normal samples were used to improve the removal of germline SNPs and sequencing errors. In this paper we focus on mutation load; a full analysis of the sequence alterations is the subject of another study.

### Statistical and survival analysis

All analyses and statistical tests were performed in Matlab (MathWorks) or R (v 2.14.1;http://www.r-project.org). All tests were two-sided and the significance level of *p* values was set to be 0.05. Survival analysis was performed on cohorts B and C combined, using Cox proportional hazard models with 10-year distant metastasis-free survival (dmfs) as end point.

## Results

### Development of an MSI signature

A cohort of 276 colorectal tumour samples (cohort A, Table 1) was analysed for their microsatellite status [microsatellite instability (MSI) or stability (MSS)] according to the local standard methodology at the originating hospital (see Methods for details); 11% (*n* = 29) of the tumours were identified as MSI (Table 1). This cohort was used for identification of genes with expression strongly associated with MSI status. Using a 10-fold cross-validation procedure, we identified a set of 64 genes (see Supplementary material, Table 1A). Optimal accuracy was reached upon classification of 57 samples as MSI by the signature and 219 samples as MSS, corresponding to a sensitivity of 93.1% and a specificity of 87.9% (Table S1) that formed the basis of a single sample-based classifier to accurately identify MSI tumours (Figure 1A). Optimal accuracy was reached upon classification of 57 samples as MSI by the signature and 219 samples as MSS, corresponding to a sensitivity of 93.1% and a specificity of 87.9% (Table 2).

**Table:** Performance of MSI gene signature: performance of MSI and MSS classification by the 64-gene signature compared to standard local hospital methodology

	Tissue	*n*	Sensitivity	Specificity	Overall accuracy
Development cohort A	Fresh	276	93.1	87.9	88.4
Validation cohort B	Fresh	132	90.3	83.2	84.8
Validation cohort D (PETTAC-3)	FFPE	625	94.3	90.1	90.6

The 64‐gene signature was validated in an independent cohort of 132 stage II colon cancer samples (validation cohort B, Table 1) that was analysed using the single sample predictor (SSP), as established in the development cohort. Performance in the validation samples showed an area under the ROC curve (AUC) of 0.942 (95% CI, 0.888–0.975) with a sensitivity of 90.3% and a specificity of 83.2% when applying the established threshold for MSS and MSI classification (Figure 1B, Table 2).

A second independent validation of gene signature was performed on a prospective cohort of FFPE tissue samples from the randomized PETACC‐3 study (cohort D, Table 1) (24). Signature read‐out in the PETACC‐3 samples showed a very high concordance with hospital‐based MSI assessment, with an ROC of 0.965 (95% CI, 0.943–0.988), which has an optimal sensitivity of 94.3% and specificity of 90.1% (Figure 1C, Table 2). Besides validating the signature in an independent prospective study, this result showed that the developed 64‐gene signature can be successfully translated to a different microarray platform and can likely be used for MSI assessment on FFPE samples.

### MSI signature and mutation frequency

In all patient cohorts, the MSI signature was able to correctly identify nearly all MSI patients (sensitivity above 92%) but they were classified as MSI by the gene signature (Figure 1)A). We hypothesized that, although these MSI‐like tumours were assessed as MSS by standard methods, they do have a deficient mismatch repair (dMMR)‐related phenotype. As such, the developed gene signature might be able to identify MSI samples but also MSS samples that harbour a dMMR phenotype (MSI‐likes).

**Figure 2:**
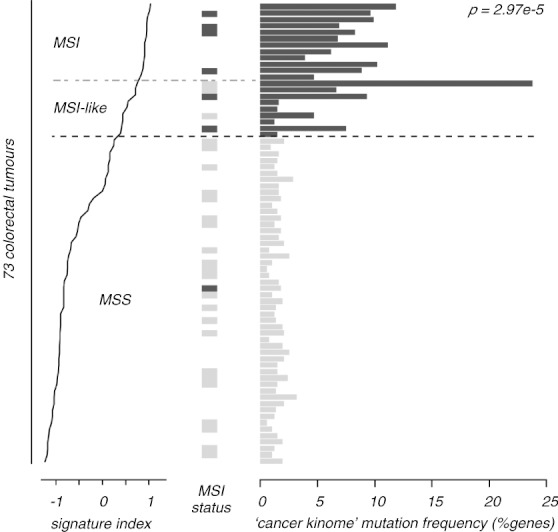
MSI and MSI‐like samples classified by the 64‐gene signature show an increased mutation frequency. Seventy‐three colorectal tumour samples were sorted according to their MSI‐signature index; the middle bar shows standard hospital‐based MSI assessment when available (light grey, MSS; dark grey, MSI) and the right barplot show the mutation frequency (% of genes mutated) of each sample in the ‘cancer kinome’ (615 genes).

To test this hypothesis, we have deep‐sequenced 73 tumour samples for their ‘cancer kinome’ (615 genes in total). The sequencing results confirmed that samples identified as MSI by the gene signature have a significantly higher mutation frequency (on average, candidate mutations were identified in 7.4% of the analysed genes) compared to MSS samples (on average, candidate mutations were identified in 1.6% of the genes) (Student's t‐test, *p* = 3.15e‐12). Importantly, further classification into MSI and MSI‐like samples indicated that the mutation frequency of the MSI‐like tumours (average 6.4%) is also significantly higher than that of MSS samples (Student's *t*‐test, *p =* 6.26e‐6) and comparable to the mutation frequency in MSI samples (8.2%) (Figure 2). This result suggests that MSI‐like tumours also harbour a dMMR phenotype, resulting in a higher mutation rate.

It is important to note that the MSI‐like patients, as identified by the signature, were not patients with a low‐grade MSI (MSI‐low) assessment by the hospital (data not shown), confirming that the MSI‐likes might be a subclass that cannot be identified by standard MSI assessment.

Investigation of activating mutations in *BRAF* showed that 64.3% of all samples classified as MSI by the gene signature harboured an activating *BRAF* mutation (36 of 56 samples with a known *BRAF* mutation status). In the MSI‐like class, 17.4% of the samples had an activating *BRAF* mutation, while the MSS classified samples were almost exclusively (98.0%) *BRAF* wild‐type (342 of 349 samples).

### Functional annotation

The association between the MSI gene signature and a dMMR phenotype was further supported by functional analysis. The results indicated that four functional annotation clusters were significantly enriched in the 64 signature genes (see Methods; see also Supplementary material, Tables S1 and S2). Annotation cluster 1 indicated that the encoded proteins of the signature are enriched with zinc‐finger domain proteins, which are often found as part of transcription, translation, DNA replication and repair machineries (30). Together with the enrichment in functional terms related to DNA binding and the nucleic acid metabolic processes (annotation cluster 2), these results are in agreement with the nature of DNA mismatch repair proteins as DNA interacting/metabolism partners that often form large complexes in the nucleus (annotation cluster 4) (31). In addition, annotation cluster 3 indicated that the signature genes are also involved in apoptosis.

### MSI‐signature and prognosis

**Figure 3:**
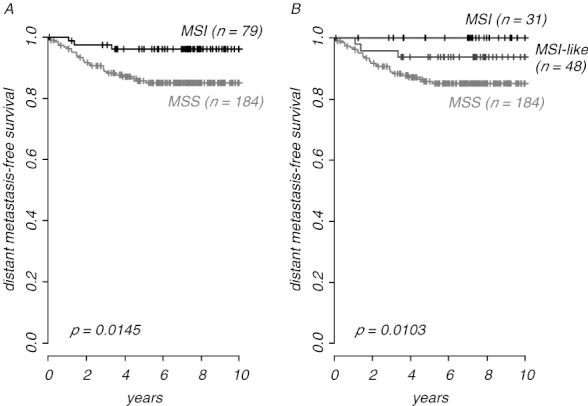
Prognostic value of the 64‐gene MSI signature in 263 stage II colorectal cancer (cohorts B and C combined). (A) Kaplan–Meier (KM) survival curves for samples classified as MSI (MSI and MSI‐like combined) and MSS by the gene signature; (B) KM curves for samples classified as MSI, MSI‐like and MSS by the gene signature. *p* values are based on log‐rank test.

The prognostic value of the 64‐gene MSI signature was tested on 263 mostly (80%) untreated stage II tumours: 132 samples from validation cohort B, plus an additional set of 131 stage II colon tumours with no available hospital‐based MSI assessment (validation cohort C, Table 1)). Patients with samples classified as MSI by the gene signature showed a significantly better distant metastasis‐free survival (DMFS) compared to patients with MSS tumours, with a hazard ratio (HR) of 0.252 (95% CI, 0.076–0.83, *p =* 0.0145) (Figure 3)A). After further subclassification into MSI, MSI‐like and MSS, the MSI‐like group also showed a significantly better survival compared to MSS samples. Interestingly, the MSI group with concordant MSI classification by signature and hospital method showed a 100% survival rate (Figure 3)B). In contrast to stage II, investigation in stage III samples (*n =* 201) showed no prognostic value of MSI/MSS classification (*p =* 0.29) (data not shown).

**Figure 4:**
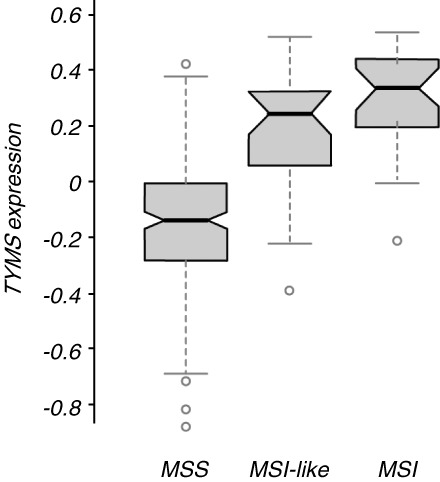
Relative gene expression levels (log_10_ scale) of thymidylate synthase (*TYMS*) in samples classified as MSS, MSI and MSI‐like by the 64‐gene signature. Samples classified as MSI showed a significant higher expression of *TYMS* compared to samples classified as MSS (*p* < 1e‐18, Student's *t*‐test) Samples classified as MSI‐like also showed a significantly higher expression of *TYMS* compared to MSS (*p =* 3.9e‐13, Student's *t*‐test).

It has been postulated that MSI patients might be resistant to 5‐FU treatment and that this resistance is associated with thymidylate synthase (TYMS) activity. We therefore investigated the expression of *TYMS* in the tumours. Samples classified as MSI showed a significant higher expression of *TYMS* compared to samples classified as MSS (cohort A, *p* < 1e‐18). Samples classified as MSI‐like showed also a significantly higher expression of *TYMS* compared to MSS (*p =* 3.9e‐13) (Figure 4).

### Technical validation of the MSI gene signature

**Figure 5:**
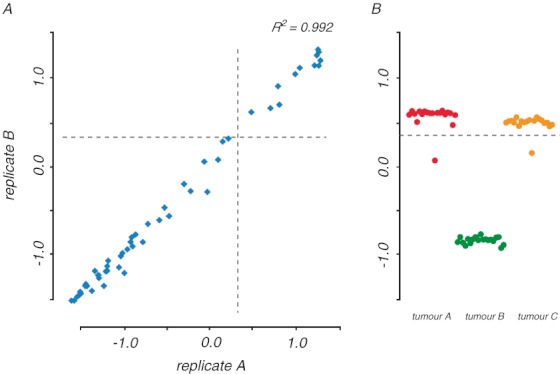
Reproducibility and precision of the 64‐gene signature. (A) Replicate analysis of 53 tumour samples shows a very high correlation in signature index. (B) Stability of the MSI signature read‐out for three representative diagnostic samples across a time period of 20 consecutive days. In both panels, the classification threshold (MSI vs MSS) is indicated by the dashed line.

The reproducibility of the MSI signature was investigated by replicate hybridization and analysis of 53 samples. MSI gene signature results were highly reproducible, with an *R*^2^ value of 0.992 (Figure 5A) and, importantly, all samples resulting in the same classification (100% concordance). Matching samples from the same patients (*n =* 60) that were either preserved as formalin‐fixed and paraffin‐embedded (FFPE) or preserved fresh in RNA‐retain were analysed to address tumour heterogeneity and technical differences between FFPE and fresh preservation. The read‐outs of MSI signature score from these two biopsies were highly correlated (*R =* 0.93) and the binary results (MSS versus MSI) were 98.4% concordant. In addition, a repeated assessment was performed for three samples over 20 consecutive days by five different technicians. Signature read‐out was stable across the 20 consecutive days, with an average standard deviation of well below 5% of the total dynamic range (Figure 5B). Of the 60 measurements, only two read‐outs resulted in a change in classification outcome (96.7% concordance). Finally, validation of the signature on the PETACC‐3 study (Figure 1C) indicated that the gene signature, which has been developed and validated on fresh‐frozen tissue samples, can be used for assessment of FFPE samples as well as fresh tissue.

## Discussion

In this report we describe the development of a 64‐gene expression signature that identifies patients with DNA mismatch repair deficiency resulting in a MSI phenotype. The signature was developed and independently validated in large sets of samples and showed high reproducibility in technical validation experiments. To our knowledge, this is the first report to describe a genomic MSI‐signature directly linked to mutation frequency, which was translated into a robust diagnostic test.

The MSI‐signature identifies patients with MSI status with high accuracy (85% and 91% accuracy in validation sets B and D, respectively) but also identifies a group of MSI‐like patients who are not recognized by traditional methods as MSI but have features similar to MSI patients, eg high mutation frequency, frequent *BRAF* mutations, high *TYMS* expression and better prognosis. This observation is in good agreement with a recently published study from the Cancer Genome Atlas (TCGA) Network that also identified a group of patients with MSI‐like features (high mutation frequency) who were classified as MSS by traditional methods (19). This is clinically relevant because these patients might be better served without adjuvant chemotherapy if they are stage II. Additionally, these result indicate that that microsatellite instability is not necessarily a good surrogate for dMMR in all patients.

Interestingly, in our study, the sample with the highest mutation frequency (23.8%) is MSI‐like by gene signature but was classified MSS by standard PCR assessment. This is again in good agreement with observations from TCGA that found that six patients with highest mutation rates were classified as MSS by standard methods. On the other hand, the single sample that was MSI by standard methods but with a strong MSS gene expression pattern in our set did not have an increased mutation rate, suggesting that this sample was incorrectly classified by standard MSI assessment (Figure 2).

The more comprehensive identification of MSI and MSI‐like patients might be explained by the fact that the read‐out of gene expression is a measurement of cellular consequences of DNA MMR deficiency in colorectal tumour, and is therefore independent of knowing the cause of the defect. At this moment, not all components of the MMR pathway in human cells are known, eg the human counterparts of *Escherichia coli MutH* and *UvrD* are not yet identified (31). Although the epigenetic silencing of *MLH1* is often observed as the main factor, other factors are known to play a role. MMR defects can be caused by any genetic or epigenetic alteration of the genes in the DNA MMR pathway. Knock‐out mouse models of *Msh2*, *Msh3*, *Msh6*, *Mlh1*, *Mlh3*, *Pms1*, *Pms2* and *Exo1* all confer a MSI phenotype 32,33. It is therefore difficult to comprehensively measure all possible sources causing MMR deficiency. Moreover, although somatic mutations in known mismatch‐repair genes might be detectable, the mutations do not always result in microsatellite instability, at least not in those microsatellites that are traditionally assessed (19). However, it is possible to summarize the cellular consequence of DNA MMR deficiency with a dominant gene expression pattern, as with the 64‐gene signature, that measures the downstream effect. The functional annotation of the 64 genes further supports the theory that the signature measures an activation that is caused by MMR deficiency, rather than the deficiency itself. Proteins with classical conserved zinc‐finger domains, DNA binding domains and associated to the nucleic acid metabolic processes were enriched in the signature. The expression signature is indicative of active DNA damage signalling, which in turn leads to cell cycle arrest and apoptosis (see Supplementary material, Table S2).

The 64‐gene signature summarizes the gene expression pattern displayed by colorectal tumours with DNA MMR deficiency, regardless of the diverse causes of this defect, and therefore might have advantages when compared to IHC or PCR methods (9). Using a gene expression signature for MSI assessment might also have technical advantages: it does not require a comparison of DNA microsatellite regions from paired normal and tumour tissues; in addition, the nature of a molecular signature builds upon the read‐out of a relatively large set of genes, resulting in robust and reproducible measurements; additionally, the MSI signature can be read out from the same tissue biopsy and in the same assay as other diagnostic signatures 20,21 minimizing sample requirements and systematic errors.

It has been well established that stage II MSI patients have better prognosis compared to patients with functional mismatch repair (34). Consistent with this knowledge, we report here that tumours predicted by the 64‐gene signature as MSI showed better distant metastasis‐free survival. While the good prognosis of MSI tumours is well documented, the value of MSI to predict response to adjuvant chemotherapy is still under investigation. Cell line models support the idea that CRCs require a functional MMR system to induce apoptosis in response to 5‐FU treatment (35). In addition, meta‐analysis of seven independent clinical studies indicated that MSI patients do not benefit from adjuvant chemotherapy with 5‐FU (12). The mechanism of action of 5‐FU is through its metabolite dUMP, which competes for the binding site of thymidylate synthase (*TYMS*), an enzyme catalysing conversion of dUMP to dTMP during DNA synthesis. The non‐responsiveness to 5‐FU therapy in MSI patients might be related to higher expression of *TYMS* in these tumours (36). In our dataset, we have confirmed this association, as MSI patients identified by the signature have high expression of *TYMS*. MSI‐like patients might present an additional population of CRC patients that are unlikely to respond to treatment with 5‐FU.

To conclude, we have developed a 64‐gene signature characterizing DNA MMR deficiency in colorectal tumours. This signature is technically robust and can be used as an alternative diagnostic tool to assess MSI status. It was implemented on a diagnostic array and validated in both fresh‐frozen and FFPE tumour samples. The results from this test provide information on the prognosis of colorectal cancer patients and aid decision making for the selection of appropriate chemotherapeutic agents.

## Author contributions

All authors were involved in writing the manuscript and in reviewing the final draft; ST, PR, RB and IS conceived experiments and study design; ST, PR, VP, MM, IA and MD performed data analysis; RS, CS, RR, UN, WM, SB and SaT were involved in sample collection, updating patient information and/or generating MSI-data; and PR, VP, IA, RS, UN, SaT, MD, RB and IS were involved in data interpretation.

## Abbreviations

5-FU: 5-fluorouracil
CRC: colorectal cancer
MMR: mismatch repair
MSI: microsatellite instability
MSS: microsatellite stability

## SUPPORTING INFORMATION ON THE INTERNET

The following supporting information may be found in the online version of this article:

Table S1 Genes in the MSI-64 gene signature

Table S2 Functional annotation: functional category analysis of the 64 genes by DAVID software
