# Supplementary material for: A robust genomic signature for the detection of colorectal cancer patients with microsatellite instability phenotype and high mutation frequency
Source: J Pathol. 2012 Oct 12;228(4):586–95. doi: 10.1002/path.4092 (PMC3532622; doi:10.1002/path.4092)
Supplement: Supplementary file 1 [file path0228-0586-SD1.doc]

### <Supplementary material>

### +A: Supplementary methods: hospital-based methods for MSI measurement

MSI status in 106 cases patients in the development cohort (cohort A) was determined by PCR amplification of six microsatellite DNA regions (D21S415, D21S1235, D12S95, D4S2948, SIT2 and BAT26) from paired normal and tumour tissues, as described previously [16]. MSI multiplex analysis, including five microsatellite DNA regions (BAT-25, BAT-26, NR-21, NR-24, Mono-27) was performed in 80 tumour samples accordingly to the local standard methodology (MSI Analysis System v 1.2, Promega). A tumour with only normal markers was defined as microsatellite-stable (MSS).

For all patients in cohort B (*n =* 132), genomic DNA of tumour and corresponding normal colon mucosa were analysed for microsatellite instability, using the Qiagen® Type-it Microsatellite PCR Kit (Qiagen, Hilden, Germany). Two mononucleotide and three dinucleotide Bethesda markers (BAT25, BAT26, D2S123, D5S346 and D17S250) were investigated. A tumour with five normal markers was defined as MSS. Irregularity in one marker was defined as low-grade microsatellite instability (MSI-L); irregularity in two or more markers was defined as high-grade microsatellite instability (MSI-H) [27].

In cohort D (PETACC-3 study), MSI status was determined using a standard panel of 10 mononucleotide and dinucleotide microsatellite loci by polymerase chain reaction (PCR) amplification of normal/tumour DNA pairs, as described [26]. Patients were classified as high (MSI-H) when 30% of the accessible markers per patient were unstable, classified as low (MSI-L) with one to two unstable markers, and stable otherwise (MSS).

The stability of each microsatellite was scored according to the absence (stable) or the presence (unstable) of mobility-shifted bands or additional bands in tumour DNA compared with normal DNA.
