# Supplementary material for: A robust genomic signature for the detection of colorectal cancer patients with microsatellite instability phenotype and high mutation frequency
Source: J Pathol. 2012 Oct 12;228(4):586–95. doi: 10.1002/path.4092 (PMC3532622; doi:10.1002/path.4092)
Supplement: Table S1 — Genes in the MSI-64 gene signature [file path0228-0586-SD2.doc]

### Table S1. Genes in the MSI-64 gene signature

| **Gene name** | **Gene description** |
| --- | --- |
| *ACSL6* | acyl-CoA synthetase long-chain family member 6 |
| *AGR2* | anterior gradient homologue 2 (*Xenopus laevis*) |
| *ARID3A* | AT-rich interactive domain 3A (BRIGHT-like) |
| *ASCL2* | achaete–scute complex homologue 2 (*Drosophila*) |
| *ASXL1* | additional sex combs-like 1 (*Drosophila*) |
| *ATP9A* | ATPase, class II, type 9A |
| *AXIN2* | axin 2 (conductin, axil) |
| *BC000986* | cDNA clone IMAGE:3446313, complete cds |
| *C10orf47* | chromosome 10 open reading frame 47 |
| *C13orf18* | chromosome 13 open reading frame 18; KIAA0226-like |
| *C20orf11* | GID complex subunit 8 homologue (*S. cerevisiae*) |
| *C20orf43* | chromosome 20 open reading frame 43 |
| *CEACAM3* | carcinoembryonic antigen-related cell adhesion molecule 3 |
| *CEACAM5* | carcinoembryonic antigen-related cell adhesion molecule 5 |
| *CEP68* | centrosomal protein 68 kDa |
| *DIDO1* | death inducer-obliterator 1 |
| *DUSP18* | dual specificity phosphatase 18 |
| *DYNLRB1* | dynein, light chain, roadblock-type 1 |
| *EP300* | E1A binding protein p300 |
| *EPDR1* | ependymin-related protein 1 (zebrafish) |
| *FBXO34* | F-box protein 34 |
| *GGA2* | Golgi-associated, *γ*-adaptin ear containing, ARF binding protein 2 |
| *GGT7* | *γ*-glutamyltransferase 7 |
| *GNG4* | guanine nucleotide binding protein (G protein), *γ*4 |
| *GPR143* | G protein-coupled receptor 143 |
| *GUCY2C* | guanylate cyclase 2C (heat-stable enterotoxin receptor) |
| *HNRNPL* | heterogeneous nuclear ribonucleoprotein L (HNRNPL) |
| *KCNK5* | potassium channel, subfamily K, member 5 |
| *KHDRBS3* | KH domain containing, RNA binding, signal transduction-associated 3 |
| *KRT23* | keratin 23 (histone deacetylase-inducible) |
| *LFNG* | LFNG *O*-fucosylpeptide 3*-β*-*N*-acetylglucosaminyltransferase |
| *LMO4* | LIM domain only 4 |
| *LOC157860* | uncharacterized LOC157860 |
| *MDM2* | p53 E3 ubiquitin protein ligase homologue (mouse) |
| *MLH1* | mutL homologue 1, colon cancer, non-polyposis type 2 (*E. coli*) |
| *OIT3* | oncoprotein-induced transcript 3 |
| *PLAGL2* | pleiomorphic adenoma gene-like 2 |
| *PPP1R3D* | protein phosphatase 1, regulatory (inhibitor) subunit 3D |
| *PRR15* | proline-rich 15 |
| *QPRT* | quinolinate phosphoribosyltransferase (nicotinate–nucleotide pyrophosphorylase (carboxylating) |
| *RNF43* | ring finger protein 43 |
| *ROCK2* | Rho-associated, coiled-coil containing protein kinase 2 |
| *RPL22L1* | ribosomal protein L22-like 1 |
| *SHROOM2* | shroom family member 2 |
| *SHROOM4* | shroom family member 4 |
| *SLC25A22* | solute carrier family 25 (mitochondrial carrier: glutamate), member 22 |
| *SMAD2* | SMAD family member 2 |
| *SMCR7L* | Smith–Magenis syndrome chromosome region, candidate 7-like |
| *SORBS1* | sorbin and SH3 domain-containing 1 |
| *STRN3* | striatin, calmodulin-binding protein 3 |
| *TCF7* | transcription factor 7 (T cell-specific, HMG-box) |
| *TFCP2L1* | transcription factor CP2-like 1 (TFCP2L1) |
| *TGFBR2* | transforming growth factor, *β* receptor II (70/80 kDa) |
| *TNFSF9* | tumour necrosis factor (ligand) superfamily, member 9 |
| *TNNC2* | troponin C type 2 (fast) |
| *TNNT1* | troponin T type 1 (skeletal, slow) |
| *TRIM7* | tripartite motif-containing 7 |
| *TSPAN6* | tetraspanin 6 |
| *UNKL* | unkempt homologue (*Drosophila*)-like |
| Unknown | unknown gene |
| *VAV3* | vav 3 guanine nucleotide exchange factor |
| *VNN2* | vanin 2 |
| *ZFP36L2* | zinc finger protein 36, C3H type-like 2 |
| *ZSWIM3* | zinc finger, SWIM-type containing 3 |

Since loss of *MLH1* is one of the most established markers for MSI assessment and *MLH1* is part of the gene signature, we wanted to confirm the performance upon exclusion of *MLH1* from the diagnostic signature. Outcomes based on the 63-gene signature without *MLH1* were almost identical to results from the full 64-gene signature, with only one sample (0.8%) that changed outcome (data not shown). This result confirmed the robustness of the signature, even without *MLH1*.
