# Supplementary material for: A robust genomic signature for the detection of colorectal cancer patients with microsatellite instability phenotype and high mutation frequency
Source: J Pathol. 2012 Oct 12;228(4):586–95. doi: 10.1002/path.4092 (PMC3532622; doi:10.1002/path.4092)
Supplement: Table S2 — Functional annotation: functional category analysis of the 64 genes by DAVID software [file path0228-0586-SD3.doc]

### Table S2. Functional annotation: functional category analysis of the 64 genes by DAVID software

| **Functional category** | **Functional term** | **Fold enrichment** |
| --- | --- | --- |
| *Annotation cluster 1* | *Enrichment score: 1.19* |  |
| SP_PIR_KEYWORDS | Zinc-finger | 2.357086 |
| SP_PIR_KEYWORDS | Zinc | 2.004079 |
| SP_PIR_KEYWORDS | Metal-binding | 1.703177 |
| GOTERM_MF_FAT | GO:0008270~zinc ion binding | 1.553891 |
| GOTERM_MF_FAT | GO:0046872~metal ion binding | 1.334464 |
| GOTERM_MF_FAT | GO:0043169~cation binding | 1.32201 |
| GOTERM_MF_FAT | GO:0043167~ion binding | 1.302684 |
| GOTERM_MF_FAT | GO:0046914~transition metal ion binding | 1.388609 |
|  |  |  |
| *Annotation cluster 2* | *Enrichment score: 0.74* |  |
| GOTERM_MF_FAT | GO:0003700~transcription factor activity | 2.549853 |
| GOTERM_BP_FAT | GO:0006357~regulation of transcription from RNA polymerase II promoter | 2.771401 |
| GOTERM_MF_FAT | GO:0030528~transcription regulator activity | 2.009639 |
| GOTERM_BP_FAT | GO:0045934~negative regulation of nucleobase, nucleoside, nucleotide and nucleic acid metabolic process | 2.810838 |
| GOTERM_BP_FAT | GO:0051172~negative regulation of nitrogen compound metabolic process | 2.772927 |
| GOTERM_BP_FAT | GO:0045892~negative regulation of transcription, DNA-dependent | 3.234043 |
| GOTERM_BP_FAT | GO:0051253~negative regulation of RNA metabolic process | 3.18044 |
| GOTERM_BP_FAT | GO:0045944~positive regulation of transcription from RNA polymerase II promoter | 3.103286 |
| GOTERM_BP_FAT | GO:0051252~regulation of RNA metabolic process | 1.587588 |
| SP_PIR_KEYWORDS | Nucleus | 1.339424 |
| GOTERM_BP_FAT | GO:0010604~positive regulation of macromolecule metabolic process | 2.015144 |
| GOTERM_BP_FAT | GO:0010557~positive regulation of macromolecule biosynthetic process | 2.200534 |
| GOTERM_BP_FAT | GO:0031328~positive regulation of cellular biosynthetic process | 2.100947 |
| GOTERM_BP_FAT | GO:0016481~negative regulation of transcription | 2.508321 |
| GOTERM_BP_FAT | GO:0009891~positive regulation of biosynthetic process | 2.070718 |
| GOTERM_BP_FAT | GO:0045893~positive regulation of transcription, DNA-dependent | 2.413667 |
| GOTERM_BP_FAT | GO:0051254~positive regulation of RNA metabolic process | 2.393595 |
| SP_PIR_KEYWORDS | Transcription regulation | 1.499065 |
| GOTERM_BP_FAT | GO:0010605~negative regulation of macromolecule metabolic process | 1.960693 |
| GOTERM_BP_FAT | GO:0010629~negative regulation of gene expression | 2.284363 |
| GOTERM_BP_FAT | GO:0006355~regulation of transcription, DNA-dependent | 1.461065 |
| SP_PIR_KEYWORDS | Transcription | 1.466492 |
| GOTERM_BP_FAT | GO:0045449~regulation of transcription | 1.327934 |
| GOTERM_MF_FAT | GO:0008134~transcription factor binding | 2.153872 |
| GOTERM_BP_FAT | GO:0006350~transcription | 1.369966 |
| GOTERM_BP_FAT | GO:0010558~negative regulation of macromolecule biosynthetic process | 2.104788 |
| GOTERM_BP_FAT | GO:0031327~negative regulation of cellular biosynthetic process | 2.052262 |
| GOTERM_BP_FAT | GO:0045941~positive regulation of transcription | 2.041346 |
| GOTERM_MF_FAT | GO:0003677~DNA binding | 1.30355 |
| GOTERM_BP_FAT | GO:0009890~negative regulation of biosynthetic process | 2.009283 |
| GOTERM_BP_FAT | GO:0010628~positive regulation of gene expression | 1.981616 |
| GOTERM_BP_FAT | GO:0045935~positive regulation of nucleobase, nucleoside, nucleotide and nucleic acid metabolic process | 1.845063 |
| GOTERM_BP_FAT | GO:0051173~positive regulation of nitrogen compound metabolic process | 1.787763 |
|  |  |  |
| *Annotation cluster 3* | *Enrichment score: 0.55* |  |
| GOTERM_BP_FAT | GO:0008219~cell death | 2.001598 |
| GOTERM_BP_FAT | GO:0016265~death | 1.987775 |
| GOTERM_BP_FAT | GO:0006915~apoptosis | 1.91249 |
| GOTERM_BP_FAT | GO:0012501~programmed cell death | 1.884319 |
|  |  |  |
| *Annotation cluster 4* | *Enrichment score: 0.50* |  |
| GOTERM_BP_FAT | GO:0006461~protein complex assembly | 2.27984 |
| GOTERM_BP_FAT | GO:0070271~protein complex biogenesis | 2.27984 |
| GOTERM_BP_FAT | GO:0065003~macromolecular complex assembly | 1.731307 |
| GOTERM_BP_FAT | GO:0043933~macromolecular complex subunit organization | 1.621576 |
